# Supplementary material for: Asynchronous magnetic resonance elastography: Shear wave speed reconstruction using noise correlation of incoherent waves
Source: Magn Reson Med. 2022 Oct 27;89(3):990–1001. doi: 10.1002/mrm.29502 (PMC9792433; doi:10.1002/mrm.29502)
Supplement: Supplementary file 1 — DATA S1 MATLAB code used to generate the numerical simulations of Figure 1 [file MRM-89-990-s002.zip › k-Wave/helpfiles/example_ewp_3D_simulation.html]

Simulations In Three Dimensions Example (k-Wave)


# Simulations In Three Dimensions Example

This example provides a simple demonstration of using k-Wave to model elastic waves in a three-dimensional heterogeneous propagation medium. It builds on the Explosive Source In A Layered Medium and Simulations In Three-Dimensions examples.

- Open the file in the MATLAB Editor
- Run the file in MATLAB

## Contents

- Running the simulation

## Running the simulation

Simulations in three-dimensions are performed in an analogous fashion to those in two dimensions. In this example, a focused velocity source from a rectangular piston is incident on a layered medium. The sensor mask is defined as a set of cuboid corners that covers the central x-y plane.

```
% define sensor mask in x-y plane using cuboid corners, where a rectangular
% mask is defined using the xyz coordinates of two opposing corners in the
% form [x1, y1, z1, x2, y2, z2].'
sensor.mask = [1 + PML_size, 1 + PML_size, Nz/2, Nx - PML_size, Ny - PML_size, Nz/2].';
```

To allow visualisation of the source elements within the grid, the source mask is assigned to the optional input parameter `'DisplayMask'`. This mask is overlaid onto the plot during the simulation. The optional input `'DataCast'` is set to `'single'` to reduce the computation time, and the `'PlotScale'` input is used to define suitable plot limits for the display of the normal and shear stress.

```
% define input arguments
input_args = {'PlotScale', [-2, 2, -0.1, 0.1], 'DataCast', 'single',...
    'PMLSize', PML_size, 'DisplayMask', source.u_mask};

% run the simulation with PML inside
sensor_data = pstdElastic3D(kgrid, medium, source, sensor, input_args{:});
```

A snapshot of the wavefield during the simulation is given below.
